# *MDM2* and *CDK4* amplifications are rare events in salivary duct carcinomas

**DOI:** 10.18632/oncotarget.12127

**Published:** 2016-09-20

**Authors:** Inga Grünewald, Marcel Trautmann, Alina Busch, Larissa Bauer, Sebastian Huss, Petra Schweinshaupt, Claudia Vollbrecht, Margarete Odenthal, Alexander Quaas, Reinhard Büttner, Moritz F. Meyer, Dirk Beutner, Karl-Bernd Hüttenbrink, Eva Wardelmann, Markus Stenner, Wolfgang Hartmann

**Affiliations:** ^1^ Department of Pathology, University Hospital Muenster, Muenster, Germany; ^2^ Department of Otorhinolaryngology, Head and Neck Surgery, University Hospital Muenster, Muenster, Germany; ^3^ Institute of Pathology, University Hospital Cologne, Cologne, Germany; ^4^ Current address: Institute of Pathology, Charité University Hospital Berlin, Berlin, Germany; ^5^ Department of Otorhinolaryngology, Head and Neck Surgery, University Hospital Cologne, Cologne, Germany

**Keywords:** salivary gland carcinoma, p53, MDM2, CDK4, HMGA2

## Abstract

Salivary duct carcinoma (SDC) is an aggressive adenocarcinoma of the salivary glands associated with poor clinical outcome. SDCs are known to carry *TP53* mutations in about 50%, however, only little is known about alternative pathogenic mechanisms within the p53 regulatory network. Particularly, data on alterations of the oncogenes *MDM2* and *CDK4* located in the chromosomal region 12q13-15 are limited in SDC, while genomic rearrangements of the adjacent *HMGA2* gene locus are well documented in subsets of SDCs. We here analyzed the mutational status of the *TP53* gene, genomic amplification of *MDM2, CDK4* and *HMGA2* rearrangement/amplification as well as protein expression of TP53 (p53), MDM2 and CDK4 in 51 *de novo* and *ex* pleomorphic adenoma SDCs.

25 of 51 cases were found to carry *TP53* mutations, associated with extreme positive immunohistochemical p53 staining levels in 13 cases. Three out of 51 tumors had an *MDM2* amplification, one of them coinciding with a *CDK4* amplification and two with a *HMGA2* rearrangement/amplification. Two of the *MDM2* amplifications occurred in the setting of a *TP53* mutation. Two out of 51 cases showed a *CDK4* amplification, one synchronously being *MDM2* amplified and the other one displaying concurrent low copy number increases of both, *MDM2* and *HMGA2*.

In summary, we here show that subgroups of SDCs display genomic amplifications of *MDM2* and/or *CDK4*, partly in association with *TP53* mutations and rearrangement/amplification of *HMGA2*. Further research is necessary to clarify the role of chromosomal region 12q13-15 alterations in SDC tumorigenesis and their potential prognostic and therapeutic relevance.

## INTRODUCTION

Salivary duct carcinoma (SDC) is an aggressive adenocarcinoma of the salivary glands, most commonly involving the parotid gland. It is one of the most aggressive salivary gland malignancies, most frequently associated with the occurrence of early distant metastasis and poor prognosis [1]. In the past few years increasing knowledge on recurrent genetic alterations of SDC evolved. In recent studies high percentages of *TP53* mutations were detected, involving around 50% of cases of *de novo* and *ex* pleomorphic adenoma SDCs [2, 3]. However, there is a large subset of *TP53* wildtype (WT) tumors which might harbor alternative alterations in the p53 regulatory network.

For decades, p53 has been a well-known tumor suppressor that is mutated or functionally inactivated in large subsets of human cancers [4]. Physiologically, transcriptional activity and stability of p53 are negatively regulated by the ubiquitin ligase MDM2, involving at least two mechanisms: a) direct blockage of the p53 transactivation domain and b) ubiquitylation-induced proteasomal degradation. Overexpression of MDM2, as found in many human tumors, is therefore capable to functionally impair p53. Inhibition of the MDM2-p53 interaction may therefore restore p53 activity and might offer opportunities for a targeted cancer therapy in tumors characterized by MDM2 overexpression [5]. In this setting, a study performed on *MDM2*-amplified well-differentiated or dedifferentiated liposarcomas (LS), which are consistently characterized by a high-level genomic amplification (frequently >15-30 copies as clusters) of sequences derived from chromosomal region 12q13-15 comprising the oncogenes *MDM2* and *CDK4*, showed that treatment with the MDM2 antagonist RG7112 activates the p53 pathway and decreases cell proliferation [6]. Most frequently, MDM2 protein deregulation occurs in tumors that retain wildtype *TP53*, but *MDM2* alterations have also been described in subsets of *TP53* mutated tumors [4, 6]. It has been shown before that genomic instability affecting the chromosomal region 12q13-15 occurs in subsets of salivary gland carcinomas [7]. Apart from these findings, results from *MDM2* transgenic mice developing mammary gland tumors suggest a crucial role for MDM2 in epithelial tumors of glandular differentiation [8].

In LS, the 12q13-15 amplicon usually, but not always, shows a co-amplification of the cell cycle regulator *CDK4* together with *MDM2* [9]. It has been shown that the small subgroup of *MDM2+/CDK4-* LS shows favorable prognostic features compared to *MDM2+/CDK4+* LS [10]. Knowledge on the *CDK4* amplification status therefore provides genomic information on the structural characteristics of the amplicon with *MDM2* being located at 12q15 and *CDK4* at 12q13.3-12 and it might add further information on an independent oncogenic mechanism apart from p53 dysfunction. Since CDK4 is the key regulator of the G1-S cell-cycle transition and drives cell-cycle progression, CDK4 inhibitors might offer new strategies for a targeted cancer therapy [11, 12].

Another gene in chromosomal region 12q13-15 frequently subject to structural alterations is *HMGA2*, encoding a high-mobility group protein. Chromosomal breaks of the *HMGA2* locus have been described in several benign mesenchymal tumors including lipomas and uterine leiomyomas [13, 14], and *HMGA2* amplification was shown in several soft tissue malignancies including liposarcomas where it is almost always co-amplified with *MDM2* [9, 15]. In salivary gland tumors, rearrangements/amplifications of *HMGA2* are well known in subsets of pleomorphic adenomas (PA) [14, 16]. Carcinomas *ex* PA have been reported to generally retain *HMGA2* rearrangements along with further gene alterations in tumor progression making *HMGA2* a potential marker for SDCs arising in PA [16].

Only very little is known about the role of MDM2 or CDK4 in SDC tumorigenesis. The major aim of this study therefore was to systematically evaluate the involvement of *MDM2* and *CDK4* alterations in SDC and to put them in context with *HMGA2* alterations known in SDC. We here report on the rare occurrence of *MDM2* and *CDK4* amplifications in a large collection of these aggressive salivary neoplasms showing a heterogeneous distribution among *TP53* wildtype tumors and those carrying a *TP53* mutation.

## RESULTS

**Figure 1:**
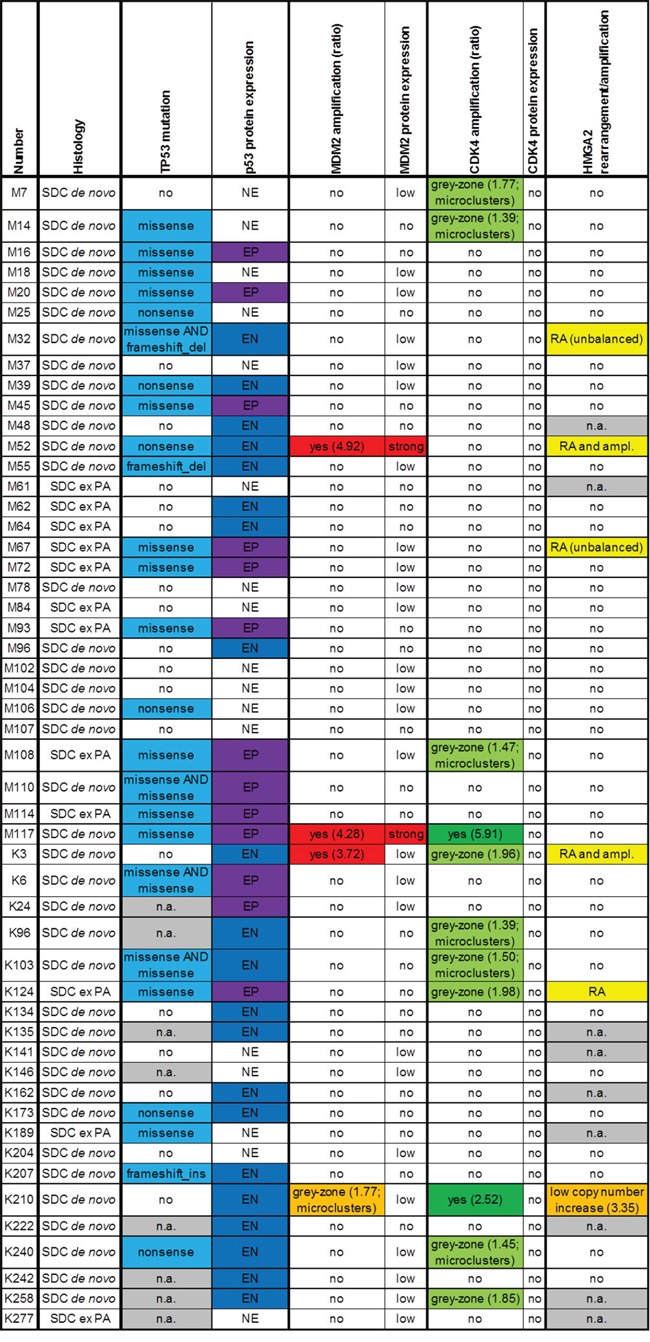
Results of immunohistochemical stainings for p53, MDM2 and CDK4 and fluorescence *in-situ* hybridization analyses for *MDM2, CDK4* and *HMGA2*, displayed for each case (PA: pleomorphic adenoma; n.a.: not available; EP: extreme positive; EN: extreme negative; NE: non-extreme, RA: rearrangement; ampl.: amplification)

**Figure 2:**
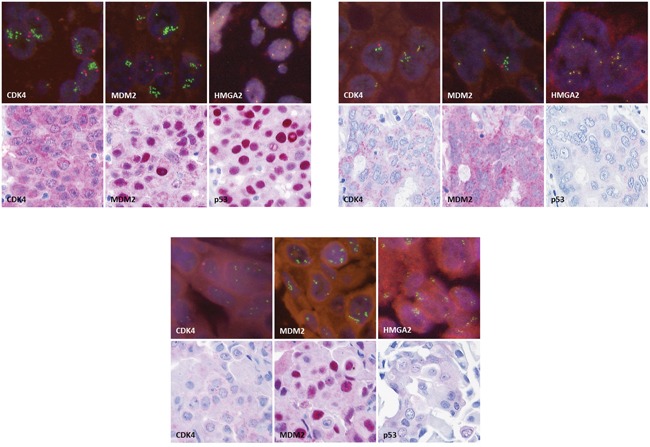
Immunohistochemical stainings for CDK4, MDM2 and p53 (original magnification 250x) and fluorescence *in-situ* hybridization for *CDK4*, *MDM2* and *HMGA2* (original magnification 630x), exemplarily for case M117 A., case K210 B. and case M52 C

51 SDC cases were analyzed for *TP53* mutational status, *MDM2, CDK4* and *HMGA2* genomic amplification as well as *HMGA2* rearrangement and p53, MDM2 and CDK4 protein expression. The clinicopathological characteristics of these 51 patients are summarized in Table 1, and the results of the mutation screen, FISH and immunohistochemical analyses are displayed in Figure 1. In Figure 2, images of immunohistochemical stainings and FISH analyses are shown exemplarily for cases M117 (Figure 2A), K210 (Figure 2B) and M52 (Figure 2C).

**Table 1:** Clinical data of the patients included in the study

Patients' characteristics	N (%)
**Patients**	51
Male	38 (74.5%)
Female	13 (25.5%)
**Age (years)**	
Mean ± SD	66.2 ± 13.0
Median	68
Minimum/Maximum	36/90
**Histology**	
SDC *de novo*	39 (76.5%)
SDC *ex* pleomorphic adenoma	12 (23.5%)
**Resection margins**	
R0	25 (49.0%)
R1	17 (33.3%)
R2	1 (2.0%)
Rx	8 (15.7%)
**pT-stage**	
pTx	1 (2.0%)
pT1	12 (23.5%)
pT2	5 (9.8%)
pT3	16 (31.4%)
pT4a	16 (31.4%)
pT4b	1 (2.0%)
**pN-stage**	
pNx	2 (3.9%)
pN0	10 (19.6%)
pN1	8 (15.7%)
pN2	30 (58.8%)
pN3	1 (2.0%)
**Extracapsular spread**	
Unknown	23 (45.1%)
Yes	15 (29.4%)
No	13 (25.5%)
**cM-stage**	
cMx	12 (23.6%)
cM0	30 (58.8%)
cM1	9 (17.6%)
**Lymphangiosis**	
Unknown	15 (29.4%)
Yes	22 (43.1%)
No	14 (27.5%)
**Hemangiosis**	
Unknown	18 (35.3%)
Yes	14 (27.5%)
No	19 (37.3%)
**Perineural invasion**	
Unknown	19 (37.3%)
Yes	22 (43.1%)
No	10 (19.6%)
**Type of parotidectomy**	
Unknown	1 (2.0%)
Lateral	4 (7.8%)
Total	24 (47.1%)
Radical	20 (39.2%)
Subtotal	2 (3.9%)
**Neck dissection**	
Yes	49 (96.1%)
No	2 (3.9%)

*TP53* mutations were detected in 25 of these 51 cases (Table 2). In 8 cases, the *TP53* mutational status could not be evaluated due to insufficient DNA content. Immunohistochemistry for p53 showed an extreme positive (EP) staining result in 13 cases, all of these cases displaying a *TP53* missense point mutation except for one case in which the *TP53* mutational status could not be evaluated due to minor DNA quality. In 8 cases with various *TP53* mutations, an extreme negative (EN) p53 immunohistochemical staining was detected and 5 cases with *TP53* mutations showed a non-extreme (NE) staining level for p53. An extreme negative (EN) p53 staining was detected in 8 cases without *TP53* mutation and in 5 further cases in which *TP53* mutational status could not be evaluated.

**Table 2:** List of *TP53* mutations detected in the analysis of 51 SDCs

No.	TP53 mutation
M14	Exon 8 p.Pro278Arg (c.833C>G)
M16	Exon 5 p.Tyr163His (c.487T>C)
M18	Exon 4 p.Pro89Leu (c.266C>T)
M20	Exon 7 p.Gly245Ser (c.733G>A)
M25	Exon 10 p.Arg342* (c.1024C>T)
M32	Exon 4 p.Pro75Leu (c.224C>T); Exon 6 p.Arg209fs (c.626_627delGA)
M39	Exon 6 p.Gln192* (c.574C>T)
M45	Exon 5 p.Asn131Tyr (c.391A>T)
M52	Exon 8 p.Arg306* (c.916C>T)
M55	Exon 6 p.Arg209fs (c.626_627delGA)
M67	Exon 8 p.Phe270Cys (c.809T>G)
M72	Exon 5 p.Lys132Glu (c.394A>G)
M93	Exon 6 p.Tyr220Cys (c.659A>G)
M106	Exon 9 p.Gln331* (c.991C>T)
M108	Exon 5 p.Arg175His (c.524G>A)
M110	Exon 7 p.Ile232Met (c.696C>G); p.Ser241Phe (c722C>T)
M114	Exon 5 p.His168Arg (c.503A>G)
M117	Exon 5 p.Ser127Phe (c.380C>T)
K6	Exon 7 p.Gly245Val (c.734G>T), Exon 10 p.Gly360Ala (c.1079G>C)
K103	Exon 8 p.Arg273Leu (c.818G>T), p.Phe270Ser (c.809T>C)
K124	Exon 7 p.Asn239Ser (c.716A>G)
K173	Exon 4 p.Tyr103* (c.309C>A)
K189	Exon 6 p.His214Arg (c.641A>G)
K207	Exon 8 p.Val274fs (c.819_820insT)
K240	Exon 4 p.Gln100* (c.298C>T)

Three out of the total 51 cases displayed an *MDM2* amplification, one case showed an *MDM2* grey-zone amplification in FISH analyses. Two cases with *MDM2* amplification strongly stained for MDM2, one *MDM2* amplified case and the grey-zone amplified case showed low MDM2 immunohistochemical staining levels. Two of the *MDM2* amplified cases synchronously carried *TP53* mutations. All *MDM2* amplifications and the *MDM2* grey-zone amplification occurred in *de novo* SDCs, none was detected in an SDC *ex* pleomorphic adenoma.

In two cases, we detected a *CDK4* amplification, one coinciding with an *MDM2* amplification and one in the setting of an *MDM2* grey-zone amplification. Nine cases showed a *CDK4* grey-zone amplification, one of these cases in the setting of an *MDM2* amplification. However, the immunohistochemical CDK4 staining was negative in all cases.

*HMGA2* FISH could be analyzed in 42 out of 51 cases. 5 cases showed rearrangement and/or amplification of *HMGA2*, among these 3 *de novo* and 2 *ex* PA SDCs. Two of the 5 cases displayed *HMGA2* rearrangement and amplification, both occurring in association with an *MDM2* amplification; the third *MDM2* (and *CDK4*) amplified case displayed no *HMGA2* alteration. One of the *HMGA2* rearrangements occurred in the setting of a *CDK4* grey-zone amplification, the two further *HMGA2* rearrangements were detected in cases without *MDM2* and/or *CDK4* amplification. One *CDK4* amplified tumor with grey-zone *MDM2* amplification showed only a low *HMGA2* copy number increase.

**Figure 3:**
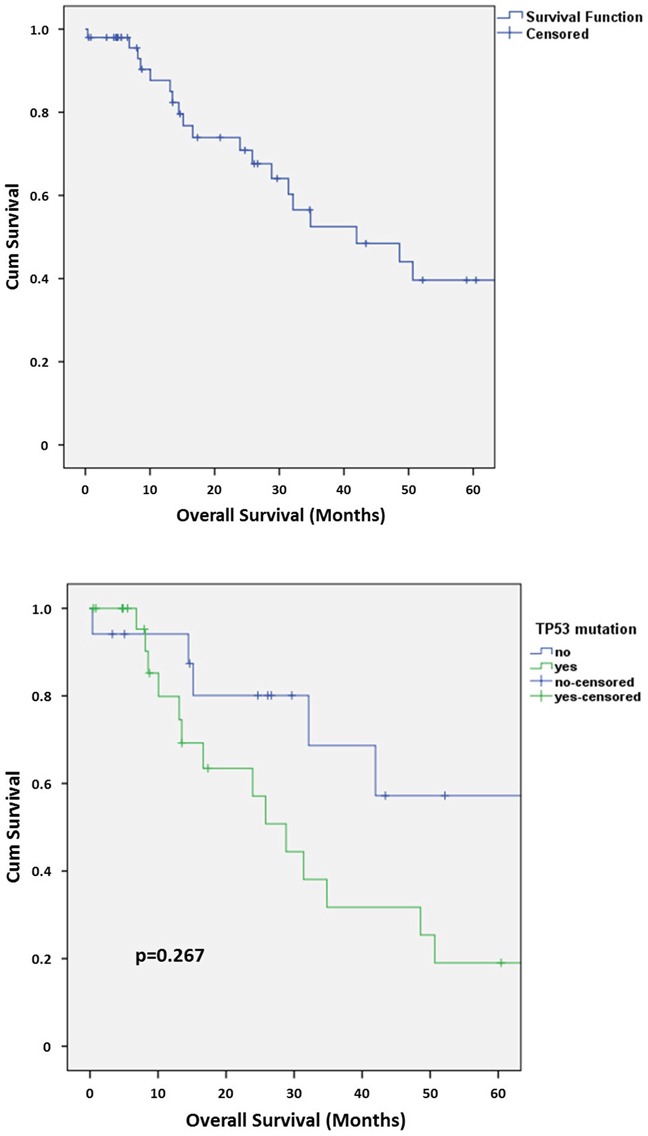
**A.** Kaplan-Meier chart of overall survival in the collection of SDCs. **B.** Kaplan-Meier chart of overall survival in the collection of SDCs depending on the presence or absence of a *TP53* mutation.

The 5-year overall survival (OS) in our collection of SDCs was 39.6% (Figure 3A). *TP53* mutated cases showed a tendency to a worse 5-year OS (20.5% with *TP53* mutation vs. 53.3% without *TP53* mutation) but statistical significance was not reached (p=0.267) (Figure 3B). No statistically significant differences in 5-year OS were detected for *MDM2* and/or *CDK4* amplified/grey-zone amplified subgroups.

## DISCUSSION

Recent genomic profiling studies confirmed a high frequency of *TP53* mutations in *de novo* and *ex* PA SDCs [2, 3], affecting about 50% of the tumors, pointing to an outstanding relevance of the p53 tumor suppressing network in the pathogenesis of these rare but highly aggressive tumors. With particular regard to the remaining 50% of SDCs harboring wildtype sequences for *TP53*, the major aim of this study was to elucidate the role of another key player within the p53 regulatory network, i.e. the ubiquitin ligase MDM2, in SDC. Due to the co-localization of *MDM2* with the cell cycle regulator *CDK4* in the chromosomal region 12q13-15 and a documented proneness of this region to genomic alterations, these regulators of cell growth and fate are frequently co-amplified in some human malignancies including a subset of soft tissue tumors [9]. Apart from providing details on the characteristics of the amplicon, knowledge of the *CDK4* amplification status might add further information on an independent oncogenic mechanism apart from p53 dysfunction, opening therapeutic options. Involvement of the chromosomal region 12q13-15 in salivary gland tumorigenesis has been documented before with the *HMGA2* gene showing rearrangements and genomic amplification in subsets of PA and SDCs *ex* PA [16]. Since *HMGA2* furthermore represents an almost constant partner of *MDM2* in the 12q13-15 amplicon known in other tumors, we complemented our analysis with the analysis of *HMGA2* to get further insight into the structure of the amplicon [9].

We detected *MDM2* amplifications in 3 cases, interestingly all classified as *de novo* SDCs. Two of these *MDM2* amplified cases showed a concurrent rearrangement/co-amplification of *HMGA2.* According to Bahrami et al. [16], who proposed alterations of *HMGA2* as a marker for SDCs *ex* PA, it could be hypothesized if these two cases might also have arisen in totally obscured PA, however, there was no evidence for a pre-existing PA, neither by histopathology nor with respect to clinical history. One of the *MDM2* amplified SDCs harbored no *HMGA2* alteration while 3 SDCs with *HMGA2* rearrangement did not show an *MDM2* amplification, implying that alterations of these two genes are obviously not strictly connected in SDCs. Thus, *MDM2* amplification appears to be a genomic alteration at least partly independent from *HMGA2* status and particularly from pathogenesis (*de novo* vs. *ex* PA) in SDC. Since only two of the cases harboring an *MDM2* amplification revealed a strong immunohistochemical staining for MDM2, immunohistochemical staining alone appears not to be a reliable marker of *MDM2* amplification in SDC. Interestingly, two of the *MDM2* amplified cases synchronously carried *TP53* mutations, while one *MDM2* amplification occurred in a *TP53*-wildtype setting. In another *TP53*-wildtype tumor a grey-zone *MDM2* amplification was detected. A very recent study on 37 SDCs revealed one *MDM2* amplified case, also in the setting of a *TP53* mutation [2]. When focusing on the MDM2-p53 regulatory connection, mechanistically, tumors with functionally inactivating mutations in *TP53* cannot be expected to substantially gain in oncogenic potential due to a concomitant amplification of *MDM2* since it usually acts as an oncogenic factor by downregulation of wildtype p53 protein. In contrast, in tumors harboring particular *TP53* mutations and retaining their wildtype *TP53* allele, *MDM2* amplification might contribute to oncogenesis by additionally disrupting the remaining functional p53 levels. Additionally, several reports argue in favor of an oncogenic role for *MDM2* independently from the p53 status [8, 17, 18]. In any case, based on our findings, in a small subset of SDCs, *MDM2* amplification obviously may serve as an alternative mechanism leading to dysregulation of the p53 network, thereby offering an option for a targeted MDM2-directed therapy [6]. With respect to documented activation steps of MDM2 through AKT1 in salivary acinar cells and mammary epithelium [19, 20], *MDM2* amplification might represent an alternative or additional mechanism in deregulating the p53 network besides well-known and pathogenetically relevant genetic alterations of the PI3K/AKT signaling pathway in SDC [3].

The immunohistochemical staining for p53 partly correlated with the *TP53* mutational status: With 48% of *TP53* mutated cases and none of the *TP53* wildtype cases showing an extreme positive (EP) staining for p53 protein expression, EP p53 staining reliably went along with a missense *TP53* mutation. 32% of *TP53* mutated cases showed an extreme negative (EN) p53 staining and 20% of *TP53* mutated cases a non-extreme (NE) p53 staining. In contrast, cases without *TP53* mutation displayed an EN p53 staining result in 44% and a NE staining in 56%. So, in summary, in case of an EN or a NE p53 staining, a reliable prediction of *TP53* mutational status is not possible in SDCs whereas an EP p53 expression is strongly suggestive of an underlying *TP53* missense mutation.

Interestingly, *TP53* mutated cases showed a tendency to a worse 5-year OS (Figure 3B), pointing to a prognostic relevance of a *TP53* mutation in SDC.

Out of the *MDM2* amplified SDCs identified in this study, two tumors showed a concomitant *CDK4* amplification, one a grey-zone *CDK4* amplification and one lacked an amplification of *CDK4*. This finding is in agreement with genomic data from a subset of liposarcomas, which frequently show a variable amplification of sequences of the chromosomal region 12q13-15. Independently from a copy number change of *MDM2*, eight tumors showed a *CDK4* grey-zone amplification (one coinciding with a *HMGA2* rearrangement), pointing to a CDK4-mediated cell-cycle dysregulation in SDC tumorigenesis. CDK4 inhibitors might therefore offer new strategies for targeted therapeutic approaches in SDCs as recently discussed for several entities [11, 12]. Interestingly, none of the *CDK4* amplified or grey-zone amplified cases showed a positive immunohistochemical staining for CDK4 (with solid staining result of the positive control), so immunohistochemical CDK4 staining obviously is not reliably suitable for the detection of *CDK4* amplified SDC. Anyway, lack of immunohistochemical CDK4 protein detection does not exclude a pathogenic role of the CDK4 oncogene and may be due to tight protein regulation beyond the threshold of staining sensitivity. Contrasting with our immunohistochemical results and potentially pointing to different characteristics of diagnostically employed antibodies, a previously reported study on five SDC cases demonstrated CDK4 protein expression in four of five cases, accompanied by MDM2 protein expression in two cases; however, *CDK4* and *MDM2* amplification status was not determined in that study [21].

As described above, in a subset of liposarcomas the small subgroup of MDM2+/CDK4- tumors shows favorable prognostic features compared to MDM2+/CDK4+ tumors [10]. In our collection of SDCs the 5-year OS was 39.6%. We could not detect any statistically significant differences of the 5-year OS in the *MDM2* and/or *CDK4* amplified/grey-zone amplified subgroups, which is probably due to the limited case numbers contained in the smaller subgroups. Larger cohorts of these aggressive tumors should be analyzed for a better understanding of a potential prognostic impact of *MDM2* and/or *CDK4* alterations.

In this study, we investigated for the first time systematically the involvement of MDM2 and CDK4 in the carcinogenesis of SDCs by analyzing the *TP53* mutational status, *MDM2* and *CDK4* amplification and *HMGA2* rearrangement/amplification as well as protein expression of p53, MDM2 and CDK4. We showed that in subgroups of SDCs, *MDM2* and/or *CDK4* amplification might play a pathogenic role, in part apparently in association with other genetic alterations. Further work is mandatory to clarify the role of *MDM2* and *CDK4* alterations in the tumorigenesis of SDCs (especially in the *TP53* mutated subgroup), the potential prognostic relevance of these alterations in SDCs and the feasibility of MDM2- and/or CDK4-directed therapeutic strategies in SDCs.

## MATERIALS AND METHODS

### Patient data and specimens

The investigation was conducted according to the Declaration of Helsinki on biomedical research involving human subjects. The retrospective study included 51 patients with newly diagnosed salivary duct carcinoma. Among these, 30 cases were derived from the University Hospital of Muenster and 21 cases from the University Hospital of Cologne (the latter having partly been included in a previous study [3]). Patients from Cologne have been treated at the Department of Otorhinolaryngology, Head and Neck Surgery at the University Hospital of Cologne between 1998 and 2011, patients from Muenster have been treated at the Department of Otorhinolaryngology, Head and Neck Surgery at the University Hospital of Muenster between 2000 and 2014. All patients were subjected to primary definitive surgery and potential adjuvant radiation according to patients' cancer stage. Tumor staging was adapted to the 7^th^ edition of the UICC TNM classification for carcinomas of the salivary glands. Patients were followed up at the outpatients department of Cologne or Muenster, respectively, at periodic visits in 3 to 6 months. At the time of analysis, 22 patients had died and 11 patients had developed a histologically confirmed relapse. Mean follow-up time was 22.2 months (range 0 to 161). The study was approved by the local Ethics Committees.

Formalin-fixed and paraffin-embedded (FFPE) material of the patients was obtained from the archives of the Departments of Pathology at the University Hospitals of Cologne and Muenster, respectively. All tumors were re-evaluated microscopically and by means of immunohistochemistry by two experienced pathologists with regard to histopathological diagnosis in accordance with WHO 2005 classification of tumors of salivary glands.

From FFPE material of all included cases, two core biopsies out of the tumor area were taken to assemble tissue microarrays (TMA).

For statistical analysis, the IBM SPSS Statistics 22 software (SPSS Inc., Chicago, IL, USA) was applied and Kaplan-Meier survival analysis and Log rank test were performed. The significance level was set at p<0.05.

### FISH analyses and immunohistochemistry

Fluorescence *in-situ* hybridization (FISH) analyses and immunohistochemical stainings were conducted on slides from TMAs. FISH analyses were performed as described previously [22, 23] using the ZytoLight^®^ SPEC MDM2/CEN 12 Dual Color Probe for assessment of *MDM2* amplification and the ZytoLight^®^ SPEC CDK4/CEN 12 Dual Color Probe for assessment of *CDK4* amplification (ZytoVision GmbH, Bremerhaven, Germany). *HMGA2* FISH analysis was performed according to a previously published assay [16, 24] using BACs RP11-662G15 and RP11-1025D9 (Life Technologies by Thermo Fisher Scientific Inc., Waltham, USA). At least 40-60 tumor cell nuclei of each tumor sample were analyzed. Amplification of *MDM2* and *CDK4* was defined as an *MDM2*/centromer 12 (CEN12) or *CDK4*/centromer 12 (CEN12) ratio ≥2.0 **or** an average number of *MDM2* or *CDK4* signals per tumor cell nucleus ≥6 **or** large clusters of *MDM2* or *CDK4* signals ≥10%, respectively. Grey-zone amplification was defined as an *MDM2*/centromer 12 (CEN12) or *CDK4*/centromer 12 (CEN12) ratio ≥1.8 **or** as microclusters of ≥5 *MDM2* or *CDK4* signals in ≥15% of tumor cell nuclei, respectively, based on modified scoring algorithms for *HER2* and *FGFR1* as published before [23, 25]. For *HMGA2* rearrangement and amplification were evaluated as published before [16].

Immunohistochemical staining was conducted on a Dako Autostainer (Dako Deutschland GmbH, Hamburg, Germany) following the manufacturer's instructions. Three μm sections were cut from the TMAs, followed by heat induced antigen retrieval in low (for p53) or high (for MDM2 and CDK4) pH buffer. For visualization LSAB method with AP/RED was used. Following antibodies and concentrations were used: p53 (1:3000, clone DO-7, Dako), MDM2 (1:100, clone IF2, Invitrogen by Thermo Fisher Scientific Inc., Waltham, USA) and CDK4 (1:100, clone DCS-31, Invitrogen). Sections were counterstained with hematoxylin and tiled with Cytoseal (Thermo Fisher Scientific Inc.). For scoring of the immunohistochemical stainings for p53, MDM2 and CDK4 only nuclear staining was rated. The staining intensity was evaluated semiquantitavely into 0, 1, 2 or 3 by comparing within the different tumor samples. To determine percentage labelling indices, all tumor cells within the cores were analyzed using high-power (400x) magnification. A sum score was calculated out of the staining intensity and the percentage labelling index, and for MDM2 and CDK4 value ranges for staining level were defined as follows: 0: no staining; >0 to 50: low staining level; >50 to 100: intermediate staining level; >100: strong staining. Sum score of p53 staining was interpreted modified according to Boyle *et al.* [26] as follows: 0: extreme negative (EN); >50: extreme positive (EP); >0 to 50: non-extreme (NE, intermediate patterns).

### Assessment of *TP53* mutational status

To complete the data on *TP53* mutational status as previously described for a smaller subset of samples [3] for the whole cohort, assessment of *TP53* mutational status was carried out as follows:

### Tumor macrodissection, DNA extraction and quantification

Sections were prepared from FFPE material and stained with hematoxylin & eosin (H&E). Six additional sections of 6 μm thickness were cut, mounted onto glass slides and used for macrodissection. In total, 1 cm^2^ tumor area corresponding to the tumor area of H&E-stained section was scraped off with a scalpel and collected into plastic tubes. Subsequently, the DNA was automatically extracted using the Maxwell DNA FFPE isolation kit on a Maxwell platform (Promega GmbH, Mannheim, Germany). Fluorometric DNA quantification was performed according to the Qubit dsDNA HS assay (Qubit 2.0, Life Technologies, Carlsbad, CA, USA).

### NGS library construction

Pre-verified multiplex PCR primer sets (summarized in Supporting Information Supplemental Table 1 A) were used to amplify the exonic region of *TP53* (customized GeneRead DNAseq Mix-n-Match V2 panel, Qiagen GmbH, Hilden, Germany). Target enrichment was processed by means of the GeneRead DNAseq Panel PCR V2 Kit (Qiagen), following the manufacturer's instructions. All purification and size selection steps were performed utilizing Agencourt AMPure XP magnetic beads (Beckman Coulter, Inc., Brea, CA, USA). End repair, A-addition and ligation to NEXTflex-96 DNA barcodes (Bioo Scientific, Austin, Texas, USA) was carried out using the GeneRead DNA Library I Core Kit (Qiagen). Amplification of adapter-ligated DNA was conducted using NEXTflex primers (Bioo Scientific) and the HiFi PCR Master Mix (GeneRead DNA I Amp Kit, Qiagen). Next generation sequencing was performed applying 12.5 pM library pools (2% PhiX V3 control) and the MiSeq Reagent v2 chemistry (Illumina, Inc., San Diego, Ca, USA).

### NGS data analysis

Fastq files were generated by the MiSeq Reporter software (Illumina) and further analyzed by means of the CLC Biomedical Genomics Workbench software (CLC bio, Qiagen). The total batch of identified *TP53* variants was filtered according to following criteria and then validated by Sanger sequencing: Hotspot artefacts and reading errors were filtered, which are recognized by high occurrence and constant frequency within the cohort. In addition, synonymous variants, germline single nucleotide polymorphisms (SNPs) and all variants below 4% allelic frequency were filtered, ending up with variants listed in Supporting Information Supplemental Table 2. Additional information on the functional impact of detected *TP53* mutations is provided in Supporting Information Supplemental Table 3.

### Sanger sequencing

Conventional Sanger sequencing was conducted according to standard procedures using the BigDye Terminator v3.1 Cycle Sequencing Kit (Thermo Fisher Scientific Inc.) and various *TP53* primer sets (summarized in Supporting Information Supplemental Table 1 B).

## SUPPLEMENTARY TABLES






