# Supplementary material for: MDM2 and CDK4 amplifications are rare events in salivary duct carcinomas
Source: Oncotarget. 2016 Sep 20;7(46):75261–72. doi: 10.18632/oncotarget.12127 (PMC5342738; doi:10.18632/oncotarget.12127)
Supplement: Supplementary file 1 [file oncotarget-07-75261-s001.pdf]

MDM2 and *CDK4* amplifications are rare events in salivary duct carcinomas

SUPPLEMENTARY TABLES

Supplemental Table 1: Primers Used for PCR Assays

See Supplementary File 1

Supplemental Table 2: Complete list of TP53 variants (non-synonymous, ≥ 4% allelic frequency)

Pat. #	*CDS mutation*	*AA mutation*	*Exon*	*Variant type*	*Allelic freq. (%)*	*Cov.*	*TCC (%)*	*Cosmic ID*
M14	c.833C>G	p.P278R	8	missense	39.3	494	50	COSM10887
M16	c.487T>C	p.Y163H	5	missense	7.8	1237	60	COSM43846
M18	c.266C>T	p.P89L	4	missense	7.1	70	50	COSM44677
M20	c.733G>A	p.G245S	7	missense	27.3	271	30	COSM6932
M25	c.1024C>T	p.R342*	10	nonsense	20.4	216	40	COSM11073
M32	c.224C>T	p.P75L	4	missense	5.1	78	70	/
M32	c.626_627delGA	p.R209fs*6	6	frameshift_del	24.5	660	70	COSM13120
M39	c.574C>T	p.Q192*	6	nonsense	16.3	502	50	COSM10733
M45	c.391A>T	p.N131Y	5	missense	45.7	129	40	COSM43533
M52	c.916C>T	p.R306*	8	nonsense	12.1	744	30	COSM10663
M55	c.626_627delGA	p.R209fs*6	6	frameshift_del	50.9	432	50	COSM13120
M67	c.809T>G	p.F270C	8	missense	5.8	753	30	COSM43621
M72	c.394A>G	p.K132E	5	missense	18.4	239	50	COSM10813
M93	c.659A>G	p.Y220C	6	missense	48.1	1082	40	COSM10758
M106	c.991C>T	p.Q331*	9	nonsense	33.7	510	50	COSM11354
M108	c.524G>A	p.R175H	5	missense	24.2	488	50	COSM10648
M110	c.696C>G	p.I232M	7	missense	23.0	413	30	/
M110	c.722C>T	p.S241F	7	missense	23.2	414	30	COSM10812
M114	c.503A>G	p.H168R	5	missense	13.5	1549	30	COSM43545
M117	c.380C>T	p.S127F	5	missense	11.9	377	50	COSM44226

(TP53 transcript ID: ENST00000269305, NM_000546)
(Variants highlighted in grey were confirmed by Sanger sequencing)
CDS: Coding DNA sequence; AA: Amino acid; Allelic freq.: Allelic frequency; Cov.: Coverage; TCC: Tumor cell content

Supplemental Table 3: Prediction on functional effects of TP53 missense mutations

See Supplementary File 2
