# Supplementary material for: MDM2 and CDK4 amplifications are rare events in salivary duct carcinomas
Source: Oncotarget. 2016 Sep 20;7(46):75261–72. doi: 10.18632/oncotarget.12127 (PMC5342738; doi:10.18632/oncotarget.12127)
Supplement: Supplementary file 2 [file oncotarget-07-75261-s002.docx]

**Supplemental Table S1: Primers Used for PCR Assays**

**A. Primer coordinates and sequences used for NGS library construction**

(TP53 transcript ID: ENST00000269305, NM_000546)

| ***Gene*** | ***Coordinates*** | ***Primer sequence (5´- 3´)*** | |
| --- | --- | --- | --- |
| TP53 | chr17:7572846-7572966 | CCTATTGCAAGCAAGGGTTC | CAGTCTACCTCCCGCCATA |
| TP53 | chr17:7572928-7573009 | GGAACAAGAAGTGGAGAATGTC | CTCCCTGCTTCTGTCTCCTAC |
| TP53 | chr17:7572962-7573081 | CCCTTCTGTCTTGAACATGAG | CTTAGGCCCTTCAAAGCAT |
| TP53 | chr17:7574001-7574120 | CAAGGCCTCATTCAGCTCT | GCATGTTGCTTTTGTACCG |
| TP53 | chr17:7576496-7576605 | GGCTAAGCTATGATGTTCCTTAGATT | GTTAAAGAGAGCATGAAAATGGT |
| TP53 | chr17:7576601-7576718 | GCATCTGTATCAGGCAAAGTC | AGTGATGCCTCAAAGACAATG |
| TP53 | chr17:7576831-7576944 | GACTGGAAACTTTCCACTTGATA | CCTCAGATTCACTTTTATCACCT |
| TP53 | chr17:7576886-7576996 | TATTCTCCATCCAGTGGTTTC | GTAAGCAAGCAGGACAAGAAG |
| TP53 | chr17:7576952-7577071 | AGAGGCAAGGAAAGGTGAT | CGCACAGAGGAAGAGAATC |
| TP53 | chr17:7577017-7577126 | GCTTCTTGTCCTGCTTGCTTA | ACTGGGACGGAACAGCTTT |
| TP53 | chr17:7577077-7577195 | CCCTTTCTTGCGGAGATTC | TGGGACAGGTAGGACCTGAT |
| TP53 | chr17:7577508-7577615 | GCTCCTGACCTGGAGTCTT | TCATCTTGGGCCTGTGTTA |
| TP53 | chr17:7578140-7578243 | CCACTGACAACCACCCTTA | CGAGTGGAAGGAAATTTGC |
| TP53 | chr17:7578180-7578301 | AGTTGCAAACCAGACCTCA | CAGGCCTCTGATTCCTCAC |
| TP53 | chr17:7578254-7578374 | ACTCCACACGCAAATTTCC | CATGAGCGCTGCTCAGATAG |
| TP53 | chr17:7578312-7578427 | CTAAGAGCAATCAGTGAGGAATC | GCCATCTACAAGCAGTCACAG |
| TP53 | chr17:7578379-7578483 | AGCTGCTCACCATCGCTAT | GTGCAGCTGTGGGTTGATT |
| TP53 | chr17:7578434-7578552 | CTCCGTCATGTGCTGTGA | TGTCTCCTTCCTCTTCCTACAGT |
| TP53 | chr17:7578535-7578603 | GCCAGTTGGCAAAACATCT | CCGTCTTCCAGTTGCTTTATC |
| TP53 | chr17:7579284-7579394 | CCAGGCATTGAAGTCTCAT | GCCCCTGTCATCTTCTGTC |
| TP53 | chr17:7579389-7579500 | TGCCCTGGTAGGTTTTCTG | AAGACCCAGGTCCAGATGA |
| TP53 | chr17:7579493-7579605 | AGCAGCCTCTGGCATTC | CCTGGTCCTCTGACTGCTCT |
| TP53 | chr17:7579576-7579697 | CAAATCATCCATTGCTTGG | CCTGAAAACAACGTTCTGG |
| TP53 | chr17:7579616-7579736 | TGGGTGAAAAGAGCAGTCA | TTCCATGGGACTGACTTTC |
| TP53 | chr17:7579733-7579847 | TTCAGGAAGTCTGAAAGACAAG | AGTCAGGAAACATTTTCAGACC |
| TP53 | chr17:7579848-7579963 | GGATCCACTCACAGTTTCCATA | GGTTGGAAGTGTCTCATGCT |

**B. TP53 primer sets used for Sanger sequencing**

| ***Primer*** | ***Exon*** | ***Primer sequence (5´- 3´)*** | ***Amplicon size*** |
| --- | --- | --- | --- |
| 78-TP53-4F | Exon 4 | CTGAGGACCTGGTCCTCTGACTGC | 360 bp |
| 79-TP53-4R | Exon 4 | GCATTGAAGTCTCATGGAAGCCAGC |  |
| 80-TP53-5F | Exon 5 | TTTCAACTCTGTCTCCTTCCTCTTCCTAC | 250 bp |
| 81-TP53-5R | Exon 5 | CAGCCCTGTCGTCTCTCCAGC |  |
| 82-TP53-6F | Exon 6 | GGCCTCTGATTCCTCACTGATTGC | 170 bp |
| 83-TP53-6R | Exon 6 | TCCTCCCAGAGACCCCAGTTGC |  |
| 84-TP53-7F | Exon 7 | AAGGCGCACTGGCCTCATCTTG | 180 bp |
| 85-TP53-7R | Exon 7 | CAGTGTGCAGGGTGGCAAGTGG |  |
| 86-TP53-8F | Exon 8 | CTGATTTCCTTACTGCCTCTTGCTTCTC | 210 bp |
| 87-TP53-8R | Exon 8 | CTCCACCGCTTCTTGTCCTGC |  |
| 88-TP53-9F | Exon 9 | GGGTGCAGTTATGCCTCAGATTCAC | 160 bp |
| 89-TP53-9R | Exon 9 | CTTTCCACTTGATAAGAGGTCCCAAGAC |  |
| 90-TP53-10F | Exon 10 | CTTACTTCTCCCCCTCCTCTGTTGC | 190 bp |
| 91-TP53-10R | Exon 10 | GCTTTCCAACCTAGGAAGGCAGG |  |

bp: Base pair
