# Supplementary material for: MDM2 and CDK4 amplifications are rare events in salivary duct carcinomas
Source: Oncotarget. 2016 Sep 20;7(46):75261–72. doi: 10.18632/oncotarget.12127 (PMC5342738; doi:10.18632/oncotarget.12127)
Supplement: Supplementary file 3 [file oncotarget-07-75261-s003.docx]

**Supplemental Table S3: Prediction on functional effects of *TP53* missense mutations**

| ***Pat.***  ***#*** | ***AA***  ***mutation*** | ***^A^ PolyPhen-2***  ***(v2.2.2r398)*** | | ***^B^ PROVEAN prediction (v1.1.3)*** | | ***^C^ SIFT***  ***(GRCh37 / Ensembl 66)*** | | ***^D^ Mutation Taster***  ***(NCBI 37 / Ensembl 69)*** | | ***^E^ Mutation Assessor***  ***(rel.3, Dec 2015)*** | | ***^F^ Combined Annotation Dependent Depletion (CADD, v.1.3)*** | |
| --- | --- | --- | --- | --- | --- | --- | --- | --- | --- | --- | --- | --- | --- |
|  |  | ***pph2***  ***prob*** | ***prediction*** | ***score*** | ***prediction*** | ***score*** | ***prediction*** | ***score*** | ***prediction*** | ***FI score*** | ***functional impact*** | ***PHRED-like***  ***C-score*** | ***category*** |
| M14 | p.P278R | 1 | probably damaging | -8.61 | deleterious | 0.000 | damaging | 103 | disease causing | 3.325 | medium | 27.8 | 1% |
| M16 | p.Y163H | 1 | probably damaging | -4.82 | deleterious | 0.004 | damaging | 83 | disease causing | 3.165 | medium | 24.2 | 1% |
| M18 | p.P89L | 0.001 | benign | -1.39 | neutral | 0.023 | damaging | 98 | polymorphism | 1.355 | low | 12.82 | 10% |
| M20 | p.G245S | 1 | probably damaging | -5.88 | deleterious | 0.003 | damaging | 56 | disease causing | 2.62 | medium | 35 | 0.1% |
| M25 | p.R342* |  | NA | -9.47 | deleterious |  | NA | 6 | disease causing |  | NA | 37 | 0.1% |
| M32 | p.P75L | 0.134 | benign | -1.31 | neutral | 0.264 | tolerated | 98 | polymorphism | 1.87 | low | 0.979 | / |
| M32 | p.R209fs*6 |  | NA | -7.61 | deleterious |  | NA |  | NA |  | NA | NA |  |
| M39 | p.Q192* |  | NA | -6.65 | deleterious |  | NA | 6 | disease causing |  | NA | 36 | 0.1% |
| M45 | p.N131Y | 1 | probably damaging | -5.16 | deleterious | 0.002 | damaging | 143 | disease causing | 3.03 | medium | 27.0 | 1% |
| M52 | p.R306* |  | NA | -4.46 | deleterious |  | NA | 6 | disease causing |  | NA | 37 | 0.1% |
| M55 | p.R209fs*6 |  | NA | -7.61 | deleterious |  | NA |  | NA |  | NA | NA |  |
| M67 | p.F270C | 1 | probably damaging | -7.15 | deleterious | 0.000 | damaging | 205 | disease causing | 2.48 | medium | 28.6 | 1% |
| M72 | p.K132E | 1 | probably damaging | -3.89 | deleterious | 0.000 | damaging | 56 | disease causing | 3.265 | medium | 29.6 | 1% |
| M93 | p.Y220C | 1 | probably damaging | -8.59 | deleterious | 0.002 | damaging | 194 | disease causing | 3.165 | medium | 28.9 | 1% |
| M106 | p.Q331* |  | NA | -8.64 | deleterious |  | NA | 6 | disease causing |  | NA | 36 | 0.1% |
| M108 | p.R175H | 0.632 | possibly damaging | -4.87 | deleterious | 0.000 | damaging | 29 | disease causing | 2.585 | medium | 25.1 | 1% |
| M110 | p.I232M | 0.92 | possibly damaging | -2.47 | neutral | 0.001 | damaging | 10 | disease causing | 2.76 | medium | 24.5 | 1% |
| M110 | p.S241F | 1 | probably damaging | -5.86 | deleterious | 0.000 | damaging | 155 | disease causing | 3.325 | medium | 27.7 | 1% |
| M114 | p.H168R | 0.865 | possibly damaging | -7.56 | deleterious | 0.008 | damaging | 29 | disease causing | 3.105 | medium | 19.21 | 10% |
| M117 | p.S127F | 1 | probably damaging | -5.68 | deleterious | 0.000 | damaging | 155 | disease causing | 3.285 | medium | 29.3 | 1% |

(A) PolPhen-2: Prediction outcome can be *benign* (0.0), *possibly damaging*, or *probably damaging* (1.0), (B) PROVEAN prediction: deleterious or neutral cutoff = -2.5, (C) SIFT prediction: tolerated or damaging cutoff = 0.05, (D) MutationTaster: The score is taken from the Grantham Matrix for AA substitutions and reflects the physicochemical difference between the original and the mutated AA (range: 0.0 to 215) but does not influence the prediction. Instead, the frequency of the respective AA exchange in known *disease causing* mutations and *polymorphisms* for the classification is used, (E) Mutation Assessor: Functional impact of a variant is described as predicted functional (*high, medium*) or predicted non-functional (*low, neutral*), (F) PHRED-like scaled C-scores rank a variant relative to all possible substitutions of the human genome. A scaled C-score ≥10 indicates that these variants are predicted to be amongst the 10% most deleterious substitutions in the human genome, ≥20 = 1% and ≥30 = 0.1%. (Variants highlighted in grey were confirmed by Sanger sequencing)
